# An Important and Often Ignored Turnaround Time in Radiology – Clinician Turnaround Time: Implications for Musculoskeletal Radiology

**DOI:** 10.5334/jbsr.1834

**Published:** 2019-08-14

**Authors:** Michael Mayer, Ronnie Sebro

**Affiliations:** 1 Department of Radiology, University of Pennsylvania, Philadelphia, PA, US; 2 Department of Orthopaedic Surgery, University of Pennsylvania, Philadelphia, PA, US; 3 Department of Genetics, University of Pennsylvania, Philadelphia, PA, US; 4 Department of Biostatistics, Epidemiology and Informatics, University of Pennsylvania, Philadelphia, PA, US

**Keywords:** Turnaround time, Musculoskeletal, Radiology, MRI

## Abstract

**Background:**

There has been an increase in routine musculoskeletal (MSK) MRI studies performed on weekends.

**Study Aims:**

First, to assess whether radiologist interpretation of routine MSK MRI studies on weekends decreases the time to when the clinician reads the radiologist’s report compared to studies performed on the weekend but interpreted the following Monday. Second, to evaluate whether reports are more likely to be read by clinicians if the MRIs are interpreted by radiologists on weekends compared to the following Monday.

**Methods:**

A random sample of 1765 patients who underwent routine MSK MRIs from January 1, 2015 to December 31, 2016 was evaluated. The radiologist turnaround times (rTATs), clinician turnaround times (cTATs) and the provider turnaround time (pTAT) were calculated. The pTAT was the sum of the rTAT and the cTAT. Fisher’s exact tests were used to compare proportions. Wilcoxon Rank Sum tests were used to compare turnaround time metrics.

**Results:**

There was no difference in the pTAT for studies performed and interpreted on the weekends compared to those performed on the weekend but interpreted the following Monday (P = 0.750). However, clinicians were significantly less likely to read the reports interpreted on the weekend compared to studies interpreted on weekdays (P = 0.001).

**Conclusion:**

Routine MSK MRI studies performed on weekends can be interpreted by radiologists on the following weekday (Monday) without affecting the time at which the clinician reads the reports and these reports are more likely to be read by clinicians if the radiologist interprets the study on a weekday.

## Introduction

There has been a steady increase in the number of imaging studies performed annually and a concomitant increase in the number of relative value units (rVUs) (a measure of clinical productivity) generated by radiologists over the past 20 years [1,2,3,4]. In addition, there has been increasing magnetic resonance imaging (MRI) scanner utilization, so that these MRI scanners are almost perpetually in use [5]. This has resulted in increased numbers of routine musculoskeletal (MSK) MRI studies performed during evenings, nights, and weekends [6,7,8,9]. An important clinical question that often arises is whether immediate interpretation of these routine MRI studies performed on weekends changes clinical management.

The first aim of the study is to evaluate whether routine MSK MRI studies performed and interpreted by radiologists on weekends have lower provider turnaround times (pTAT’s) compared to routine MSK MRI studies performed on weekends but interpreted the following Monday. The second aim was to assess whether MRI reports from studies performed on the weekend were more likely to be read by clinicians if these studies were interpreted by radiologists on weekends compared to those interpreted the following Monday.

## Materials and Methods

The study was approved by the local institutional review board. The need for signed informed consent was waived.

### Turnaround times

There are several turnaround times that are often monitored in radiology departments as part of quality improvement and assurance programs [10,11,12,13]. The time from the moment the study is ordered by the clinician to the moment the MRI is performed and becomes available to the radiologist to interpret is defined for the purposes of this manuscript as the system turnaround time (sTAT), which is not provider (radiologist or clinician) dependent. The time from the MRI study becoming available to the radiologist for interpretation to the time the radiology report becomes available to the clinician is defined as the radiologist’s turnaround time (rTAT) [13]. The time from when the radiology report is available to the clinician to the time the clinician reads the report is defined as the clinician’s turnaround time (cTAT). The provider turnaround time (pTAT) is defined as the sum of the radiology turnaround time (rTAT) and the clinician turnaround time (cTAT). Therefore,

Equation 1 {\rm{pTAT}}\ =\ {\rm{rTAT}}\ + {\rm{cTAT}}

**Figure 1:**
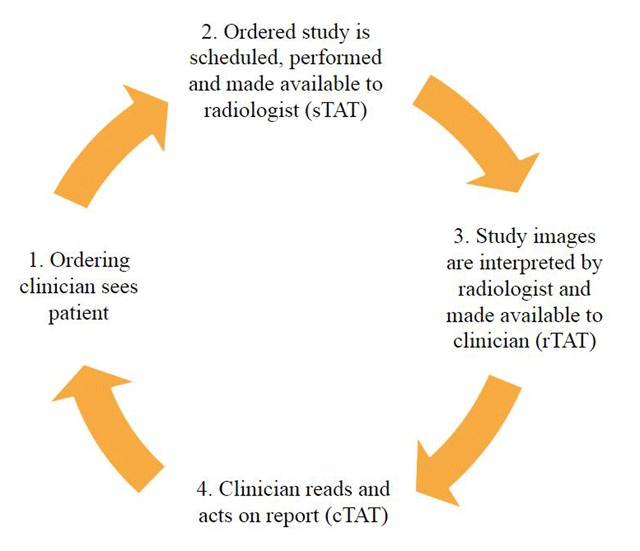
Diagram showing the sequence of events from when a clinician orders an imaging study to when the clinician reads the study.

Finally, the time for the entire process (the time from when the clinician orders the study to the time the clinician reads the radiology report) is defined as the functional turnaround time (fTAT) (Figure 1).

At our institution, the EPIC electronic medical record (EMR) is utilized (Epic Systems Corp., Verona, WI). This EMR digitally records the date and time the study was ordered, the date and time the radiologist finalized his/her report, and the date and time any provider, including the ordering provider, reads that report. The rTAT, cTAT, pTAT and fTAT can therefore be rapidly obtained from the EPIC EMR for any study.

### Patients/Studies

The EMRs of 2243 retrospectively randomly identified patients who underwent routine MSK MRIs of the cervical spine, thoracic spine, lumbar spine, pelvis, hip, ankle, foot, shoulder, elbow, wrist and hands were analyzed. For each MRI included in the study, the day the MRI was performed, as well as the day the corresponding radiology report was finalized, were recorded. The rTAT, cTAT, and pTAT were then calculated for each MRI study.

The patient’s age and sex at the time of the MRI, as well as the MRI study object (cervical spine, thoracic spine, lumbar spine, pelvis, hip, ankle, foot, shoulder, elbow, wrist and hands), and the ordering clinician’s specialty were also recorded. We evaluated up to 200 consecutive patients for each MRI study type that were imaged at an academic tertiary healthcare center between September 1, 2015 and December 31, 2016. These imaging studies were followed for 2 years to evaluate whether the imaging reports were read by the ordering provider.

MRI studies were included if they were read by an attending radiologist only, because the date and time of the preliminary report issued by residents/fellows were not routinely recorded, and because it was possible for an ordering clinician to read the resident’s preliminary report and never read the official radiologist’s report. All STAT and expedited studies were excluded. MRI studies were excluded if the attending radiologist noticed abnormal findings and called the ordering clinician or sent an electronic message to notify the clinician of these abnormal findings (N = 29). Finally, studies were excluded if they were ordered by providers outside of the healthcare system, since there was no reliable way to identify the date and time that the study report was read, because some of these providers did not have electronic access to the EMR. This resulted in 1765 studies for evaluation.

### Statistics

We hypothesized that routine MSK MRI studies performed and interpreted on a weekend day (Friday after 5 pm, Saturday or Sunday), henceforth referred to as weekend-weekend studies, would have at least 1 day decreased pTAT compared to studies performed on a weekend day but interpreted the following Monday, henceforth referred to as weekend-weekday studies. A sample size of 198 MRI studies (99 weekend-weekend and 99 weekend-weekday) has 80% power to detect a one-day difference between the mean pTAT of weekend-weekend studies compared to weekend-weekday studies, assuming a pTAT standard deviation of 2.5 days and a 1:1 ratio of weekend-weekend to weekend-weekday MRI studies. Because we suspected that the radiologist’s report is usually read by the clinician at the patient’s next clinical visit, and because several patients are often only seen by their clinicians annually or biannually, MRI studies that were at least 1-year-old were chosen to ensure sufficient time for follow-up. Data from studies performed and interpreted on weekdays (Monday, Tuesday, Wednesday, Thursday or Friday before 5 pm), henceforth referred to as weekday-weekday studies were also included for comparison.

Summary statistics were calculated for clinical and demographic variables. Fisher’s exact tests were used to compare proportions, t-tests were used to compare demographic measures and Wilcoxon-Rank Sum tests were used to compare quantitative measures. Statistics were calculated using Rv3.4 [14]. All test statistics were two-sided and P-values <0.05 were considered statistically significant.

## Results

There were 567 different ordering clinicians and 35 different interpreting radiologists. The majority of the routine MSK MRI studies were ordered by internists (40.8%; n = 720), whereas emergency medicine clinicians (2.4%; n = 43) least frequently ordered routine MSK MRI studies (Table 1).

**Table 1:** Demographics.

	Weekend-Weekday (N = 131)	Weekend-Weekend (N = 157)	Weekday-Weekday (N = 1477)	P-value^1^	P-value^2^	P-value^3^

Patient age in years (SD) [median]	47.34 (16.7) [46.0]	47.52 (17.6) [49.0]	49.87 (16.3) [51.0]	0.929	0.098	0.111
Sex (% male)	51 (38.9%)	66 (42.0%)	606 (41.0%)	0.631	0.711	0.799
MRI type				0.051	0.117	<0.001
Ankle	12 (9.2%)	15 (9.6%)	163 (11.0%)			
Cervical spine	12 (9.2%)	5 (3.2%)	136 (9.2%)			
Elbow	7 (5.3%)	10 (6.4%)	102 (6.9%)			
Foot	17 (13.0%)	22 (14.0%)	130 (8.8%)			
Hand	3 (2.3%)	6 (3.8%)	50 (3.4%)			
Hip	14 (10.7%)	26 (16.6%)	120 (8.2%)			
Lumbar spine	15 (11.5%)	9 (5.7%)	135 (9.1%)			
Pelvis	17 (13.0%)	8 (5.1%)	126 (8.5%)			
Knee	6 (4.6%)	16 (10.2%)	94 (6.4%)			
Shoulder	6 (4.6%)	13 (8.3%)	148 (10.0%)			
Thoracic spine	16 (12.2%)	15 (9.6%)	133 (9.0%)			
Wrist	6 (4.6%)	8 (5.1%)	140 (9.5%)			
Ordering clinician specialty				0.005	0.012	0.006
Emergency Medicine	2 (1.6%)	8 (5.7%)	34 (2.3%)			
Family Medicine	8 (6.4%)	25 (17.7%)	141 (9.5%)			
Internal Medicine	52 (41.6%)	56 (39.7%)	645 (43.7%)			
Orthopedics	30 (24.0%)	34 (24.1%)	445 (30.1%)			
PMR	22 (17.6%)	11 (7.8%)	129 (8.7%)			
General Surgery	11 (8.8%)	7 (5.0%)	83 (5.6%)			

PMR – Physical Medicine and Rehabilitation. SD – Standard deviation.

The mean (standard deviation) fTAT was 17.7 (74.2) days with a range of 0.01–809.6 days. The median pTAT for weekend-weekend studies was no different from weekend-weekday studies (P = 0.750) (Table 2). The radiologist’s reports from routine MRI studies were more likely to be read by clinicians for weekend-weekday studies (106/131; 78.6%) than for weekend-weekend studies (99/157; 63.1%) (P = 0.001). The rTAT accounted for a median 1.3% of the fTAT for weekday-weekday studies; 7.5% for weekend-weekend studies, and 14.5% for weekend-weekday studies. The cTAT accounted for a median 5.0% of the fTAT for weekday-weekday studies; 20.9% for weekend-weekend studies, and 2.8% for weekend-weekday studies.

**Table 2:** Turnaround times by the day of week the study was performed and interpreted by the radiologist (weekday versus weekend).

	Weekend-Weekday (N = 131)	Weekend-Weekend (N = 157)	Weekday-Weekday (N = 1477)	P-value^1^	P-value^2^	P-value^3^

**Radiologist turnaround time (rTAT) in days (median)**	2.5	0.8	0.13	<0.001	<0.001	<0.001
**Clinician turnaround time (cTAT) in days (median)**	0.3 {106/131}	1.9 {99/157}	0.6 {1171/1477}	<0.001 {0.001}	0.449 {0.736}	<0.001 {<0.001}
**Provider turnaround time (pTAT) in days (median)**	2.8	2.8	0.9	0.750	<0.001	<0.001

Number of referring clinicians that read the report/Number of reports. P-values No brackets – P-value from Wilcoxon-Rank sum test. {} – P-value comparing proportion of referring clinicians that read reports. ^1^ P-value from test comparing weekend-weekday studies to weekend-weekend studies. ^2^ P-value from test comparing weekend-weekday studies to weekday-weekday studies. ^3^ P-value from test comparing weekend-weekend studies to weekday-weekday studies.

The subgroup analysis of the cTAT by ordering clinician specialty shows that the cTAT was generally longer for weekend-weekend studies than for weekend-weekday or weekday-weekday studies (Table 3). We found that interventionalists (Physical medicine and rehabilitation (PMR) and Orthopedists) were more likely to read the radiology reports from routine MSK MRI studies than non-interventionalists (P < 0.001). Emergency physicians (36.4% [16/44]) and general surgeons (45.5% [46/101]) were the least likely to read the radiology reports.

**Table 3:** Clinician turnaround time (cTAT) in days by ordering clinician specialty.

	Weekend-Weekday (N = 131)	Weekend-Weekend (N = 157)	Weekday-Weekday (N = 1479)	P-value^1^	P-value^2^	P-value^3^

Emergency Medicine (N=)	28.4 (39.9) [11.22]	2.29 (2.3) [1.58]	14.90 (57.3) [0.23]	0.374 [0.700]	0.633 [0.389]	0.276 [0.429]
Family Medicine (N=)	2.01 (4.07) [0.14]	35.06 (142.2) [1.71]	25.88 (106.2) [0.58]	0.277 **[0.008]**	0.012 [0.377]	0.770 **[0.019]**
Internal Medicine (N=)	3.49 (7.93) [0.13]	19.97 (73.8) [1.89]	11.11 (50.19) [0.63]	0.346 **[<0.001]**	0.009 [0.059]	0.610 **[0.002]**
Orthopedics (N=)	28.96 (120.3) [0.65]	31.88 (87.87) [2.58]	8.72 (44.72) [0.29]	0.896 **[0.025]**	0.275 **[0.009]**	0.089 **[<0.001]**
PMR (N=)	70.07 (198.1) [1.82]	1.51 (1.08) [1.48]	30.02 (109.1) [0.74]	0.330 [0.776]	0.565 [0.116]	**0.005** [0.470]
General Surgery (N=)	41.65 (92.5) [0.02]	0.96 (0.97) [0.82]	12.92 (50.8) [0.80]	0.381 [0.571]	0.528 [0.336]	0.050 [0.829]

Means (Standard Deviations). [Medians]{Proportions of referring clinicians that read report}. P-values No brackets – P-value from t-test with unequal variances comparing means. [] – P-value from Wilcoxon-Rank sum test. {} – P-value comparing proportion of referring clinicians that read reports.

Of the 2243 studies initially evaluated before exclusion, 28 (1.2%) had findings that required communicating an electronic notice (pulmonary nodules [N = 1; 0.04%], adnexal lesions [N = 1; 0.04%], spinal canal stenosis [N = 2; 0.09%], unknown fractures [N = 12; 0.5%], mass/progression of metastatic disease [N = 7; 0.3%], infection/inflammatory change [N = 2; 0.09%], anterior cruciate ligament (ACL)/achilles/ankle ligament tear [N = 3; 0.1%]), and one study had findings that required a telephone call (0.04%). These 29 studies were excluded from the study. The clinician read the report in 17 (58.6%) of these studies. Of these 29 studies with notifications, three (33.9%) of the notifications were done by the radiologist on Monday, five (17.2%) on Tuesday, eight (27.6%) on Wednesday, one (3.4%) on Thursday, eight (27.6%) on Friday and one (3.4%) on Saturday. Approximately 41.2% (8/17) of these notifications were read by the clinician on Monday, 17.6% (3/17) on Tuesday, 17.6 (3/17) on Wednesday, 23.5% (4/17) on Thursday, 0% (0/17) on Friday and 0/17 on Saturday or Sunday.

## Discussion

The data show that there was no difference in the pTAT for MRI studies performed and interpreted on the weekends compared to those performed on the weekend but interpreted the following Monday. However, the cTAT was a median 1.6 days longer for studies performed and interpreted on the weekends compared to studies performed on the weekend but interpreted on Monday. The study suggests that provider turnaround times (both rTAT and cTAT) are influenced by the part of the week the study was performed on (weekdays versus weekends). The data also show that the reports of studies interpreted on the weekends were less likely to be read than the reports of studies interpreted on weekdays. This is perhaps due to the ordering physicians opening these images for self-interpretation or simply being overwhelmed by the number of messages from the weekend in the system.

Our study has significant clinical implications. At our institution, interpretation of routine MSK MRI studies on weekends did not shorten the time to when the ordering clinician was able to read the report, which is the most important reason for interpreting these studies over the weekend. The reports were mostly read by clinicians the following Monday rather than over the weekend. The study also suggests that the reports from MRI studies that were interpreted over the weekend were less likely to be read by clinicians. If the clinician never read the report, then he/she would be less likely to be able to act on the findings of that report, which defeats the entire purpose of performing the imaging study. A simple solution would be to send an automatic reminder to the ordering physician if the report from the weekend is not read after a certain number of days, although more studies are required to further investigate this. Our data suggest that routine MRIs should probably be interpreted by radiologists on weekdays to increase the likelihood that clinicians read these reports.

Utilizing incentive plans for radiologists that are focused on rapid rTATs overlooks other significant factors that impact effective, efficient clinical care. For example, the timeline of clinicians reviewing studies differed depending on whether the radiologist interpreted the study on a weekday or weekend. The problems with incentivizing rapid rTATs become clear. The reality is that radiology reports have little impact on clinical care until the referring clinician reads the radiology report and acts. Consequently, the effort to achieve a rapid turnaround time for routine MSK MRI studies by working long hours and weekends seems fruitless, because it does not affect how quickly clinicians actually review the radiologists’ reports. We suspect clinicians read the radiology reports while they are on clinical service during the weekdays, but don’t read radiology reports during the weekends – leading to the approximate two-day delay in cTAT. We also suspect that if a clinician receives too many reports over the weekend when they return to service on Monday, some of these reports may be ignored. However, more research is required to confirm this assertion.

Other factors need to be considered prior to adopting other workplace/workflow strategies. For example, staffing may be short on Mondays, requiring radiologists to work and interpret routine studies on weekends. Some patients may want access to their reports immediately. There are also medicolegal implications to be considered. Also, there is increased pressure to optimize the utilization rates of MRI machines, including on nights and weekends [15]. Although these MRI studies are routine, critical findings may be present and undiscovered for 2 days. Further research is required to better understand how to optimize the workflow in the radiology department and patient care simultaneously. Our results suggest that an important but neglected metric is the cTAT. The radiology report has no clinical importance until the requesting clinician reads it. Incentives for clinicians may optimize the cTAT to ensure that clinicians can act timely on the radiologist’s reports.

Anecdotally, some musculoskeletal radiologists have opined that their reports are not generally read by orthopedists. Our data show that orthopedic surgeons were amongst the specialists that most frequently read the radiologists’ reports. While several orthopedists are able to review their own imaging studies, the data suggests they rely on the radiologist’s report to confirm findings and to exclude other incidental findings. Finally, not all reports of requested routine MSK MRI scans were read by the clinicians, which calls the indication of these scans into question.

### Study Limitations

This study has a few limitations. The study was retrospective and based on extracting data from hospitals affiliated with a single healthcare system. Clinician data and behavior may not accurately reflect national trends, and may only be applicable to our healthcare system. Some clinicians may believe they have the confidence to interpret imaging studies independently, prior to the radiologist issuing a report. Some of these clinicians believe that the radiology report has little or no added value to their own interpretation, and will not review the radiology report. It is difficult to differentiate the clinicians who are able to interpret the MRI’s themselves and thus do not read the radiology report from the clinicians who didn’t look at the scan nor the report. There might be errors in the documentation of the exact time an MRI study is completed and made available to the radiologist, so small errors may be possible in the calculation of radiologist’s turnaround time. Not all healthcare systems have electronic medical records, so these results are probably not generalizable to these systems, however we suspect the clinician turnaround times reported are probably lower than those in a paper-based, non-electronic medical record system. Finally, even though the study was appropriately powered to investigate the primary aim, the small sample size may limit the true generalizability of the results of this study to other healthcare centers.

## Conclusion

Routine MSK MRI studies performed on weekends can be interpreted by radiologists on the following weekday (Monday) without affecting the time at which the clinician reads the reports. These reports are more likely to be read by clinicians if the radiologist interprets the study on a weekday.

## Abbreviations

Relative value units (rVUs); System turnaround time (sTAT); Radiologist’s turnaround time (rTAT); Clinician’s turnaround time (cTAT); Provider turnaround time (pTAT); Functional turnaround time (fTAT).
